# Caesarean section in a semi-rural hospital in Northern Namibia

**DOI:** 10.1186/1471-2393-7-2

**Published:** 2007-03-08

**Authors:** Jeroen van Dillen, Tarek Meguid, Vera Petrova, Jos van Roosmalen

**Affiliations:** 1 Department of Obstetrics and Gynaecology, Leiden University Medical Centre, The Netherlands; 2 Department of Obstetrics, Kamuzu Central and Bottom Hospital, Lilongwe, Malawi; 3 Department Obstetrics and Gynaecology, Onandjokwe Lutheran Hospital, Ondangwa, Namibia

## Abstract

**Background:**

Increasing caesarean sections rates (CSR) are a major public health concern and the prevention of the first caesarean section, which often leads to repeat operations, is an important issue. Analyzing caesarean sections can help to identify factors associated with variations in CSR and help to assess the quality of clinical care.

**Methods:**

In a retrospective observational study, during a two year period, indications of 576 caesarean sections were analyzed using intra-operative internal pelvimetry and a record keeping system in a semi-rural hospital in Northern Namibia.

**Results:**

Most caesarean sections were done for dystocia (34%) followed by repeat caesarean section (31%). The true conjugate (distance between the promontorium to mid pubic bone) was significantly smaller in these recurrent indication groups when compared to non recurrent indications.

**Conclusion:**

In this rural hospital the introduction of Delee Pelvimetry and a caesarean section record keeping system was found to be a simple and cheap method to analyse indications for caesarean sections, which may help in reducing unnecessary caesarean sections.

## Background

Caesarean section rates (CSR) have been increasing ever since the 1970's. The debate concerning this public health issue has recently led to the introduction of a new indication for the operation: the 'no indicated risk' caesarean [1]. Although the rise in caesarean sections is a global phenomenon, international and national variations are considerable. In England, Wales and Northern Ireland, the first national caesarean section survey in 2001 aimed to determine factors associated with these variations and to assess the quality of obstetric care during labor. This national audit was anticipated to initiate continued local audits [2].

Reducing the number of first caesarean sections is the most important issue. One of the most common indications for caesarean section is dystocia. Dystocia may be caused by cephalopelvic disproportion and pelvic inlet contraction will often be found [3,4]. Many studies have looked into selection of high risk women for dystocia, evaluating external pelvimetric measurements [5-7] or X-ray and magnetic-resonance pelvimetry [8,9]. For singleton vertex presentations at term (the 'no indicated risk' group) the baby's head remains the best pelvimeter and every woman deserves a trial of labor [10-12].

In Namibia, antenatal policy includes manual pelvic assessment at 36 weeks gestation for all primiparous women. Because of its poor sensitivity to predict dystocia, it has been recently proposed to abolish this routine and actively promote (primiparous) women to deliver in hospital [13]. We report our experience with a local caesarean section audit using internal pelvimetry in a semi-rural hospital in Northern Namibia.

## Methods

### Study site and population

Onandjokwe Lutheran Hospital is a 450 bed district hospital which also serves as referral hospital for the region. In 2002, the department of obstetrics and gynaecology (O&G) was staffed by 4 doctors (two foreign specialists, two foreign medical doctors) and 34 nurses including 13 registered/enrolled midwives. An effort has been made to improve the quality of care by using the partograph developed by the World Health Organization (WHO), standardised protocols and regular in-service-training sessions [14]. The hospital has good quality ultrasound equipment, a maternity waiting home on the hospital premises and offers comprehensive emergency obstetric care (EOC) [15]. The distribution of blood for transfusion, however, is centrally regulated in Windhoek. Occasional shortages of blood do occur. Forceps were not used during the study period.

The anesthetic department was staffed by two foreign specialists providing regional or general anesthesia. In general, regional (spinal) anesthesia was used for caesarean section.

Since 1999, the Misgav Ladach method for caesarean section is used [16]. This method uses the Joel Cohen incision and is recommended by the National Institute for Clinical Excellence [17]. During caesarean section, the true conjugate (conjugata vera) is measured using DeLee's internal pelvimeter. After hysteroraphy and just before replacing the uterus in the abdominal cavity, the true conjugate is measured by placing one arm at the midpoint of the sacral promontorium and the other arm approximately 0,5 cm down the midline of the upper posterior border of the pubic symphysis [4]. In the absence of a pelvimeter, King [18] advises to use a steel ruler.

An additional 'caesarean section record book' is kept in the theatre department since October 1997. The doctor performing the operation, which in almost all cases was also the doctor who determined the indication for caesarean, was responsible for filling the record book.

### Study objective and design

The primary objective of this study was to evaluate indications for caesarean sections in this setting. Secondly, we used the internal pelvimetry results for analyzing the indications.

All caesarean sections performed from January 1^st ^2001 – December 31^st ^2002 are included. Information from the 'caesarean section record book' was used and data collected included: date of delivery, indication for caesarean section, true conjugate (conjugata vera), Apgar score and sex of neonate, additional comments (including bilateral tubal ligation, hysterectomy).

Indications for caesarean section were categorised as follows: dystocia, repeat caesarean section (+ number of repeat caesarean), malpresentation (including: breech, twin, arm/compound and other presentation), ante partum hemorrhage, cord prolapse, fetal distress, pre eclampsia, and other indications. Dystocia combines the following indications: delay first stage, delay second stage, failed trial of vacuum, discoordinate uterine action and cephalopelvic disproportion (CPD). Only the primary indication as recorded by the operating doctor was used. In general, a diagnosis of dystocia is only made when the action line of the partograph has been passed and labor augmented with oxytocin. Data has been analysed using Microsoft Excel^®^, Windows^® ^98. For normal distributions, student t-test was used. Statistical significance was assumed if p < 0.05.

In rural Namibia the management board of the hospital is responsible for approving research projects. In this case, the management board of Lutheran Medical Services, Onandjokwe Hospital, approved the study.

## Results

**Figure 1:**
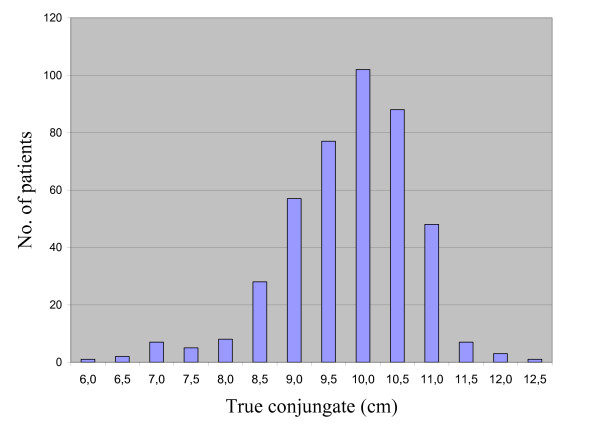
Distribution of True Conjugate (n = 434).

During the study period, 576 caesarean sections were performed in 7.321 births (CSR 7,9%). Out of the 599 neonates, 266 (44%) were female and 318 (53%) male. Sex was not recorded in 15 cases (3%). The true conjugate was measured in 434 women (75,3%) with a mean of 9,8 cm (range 6–12,5 cm; standard deviation (sd 1,0) (figure 1). Of 142 cases were true conjugate was not measured, 71 also had bilateral tubal ligation done and in 10 hysterectomy was performed. The mean Apgar score after one minute was 7,7 (range 0–10; sd 2,2; n = 563) and after five minutes 9,1 (range 0–10; sd 1,8; n = 563).

Indications for operations are shown in table 1. Most caesarean sections were performed for dystocia: 193 (33.5%) followed by repeat caesarean section: 177 (30.7%). For repeat sections: 108 were second, 52 were third, 16 were fourth and one was a fifth caesarean section. Elective repeat caesarean section was performed in 124 cases (70%), 45 (25%) were failed trials of scar, and eight (5%) were due to fetal distress (four), malpresentation (three) or (pre) eclampsia (one). Of all second caesarean sections, elective repeat operations were performed in 53 women (50%).

**Table 1:** Indication Caesarean Section, Number, Mean True Conjugate (standard deviation) and Mean Apgar scores at 5 minutes (standard deviation)

**Indication**	**Number (%)**	**True Conjugate**		**Apgar 5**
			
		**measured %**	**(cm)**	
Dystocia	193 (33.5)	83,4	9.6 (1.0)^2^	9.3
Repeat Caesarean Section	177 (30.7)	64,4	9.5 (0.9)^2^	9.6
- Elective repeat^1^	132 (22.9)		9.5 (1.0)^2^	9.6
- Failed trial of scar	45 (7.8)		9.5 (0.8)^3^	9.5
Malpresentation	55 (9.5)	76,4	10.1 (0.8)	9.6
Fetal Distress	50 (8.7)	82,0	9.9 (0.8)	8.1 ^2^
Ante Partum Hemorrhage	40 (6.9)	75,0	10.4 (0.6)	7.2 ^2^
Cord Presentation	18 (3.1)	77,8	10.1 (1.1)	7.9 ^2^
Pre eclampsia	9 (1.6)	100	10.3 (0.4)	8.3
Other	34 (5.9)	67,6	10.4 (1.1)	8.5

**Total**	**576**	**75,3**	**9.8 (1.0)**	**9.1**

^1 ^Elective repeat caesarean section (132) including 8 cases where it is unknown if the women was in labor: fetal distress (4), malpresentation (3) and preeclampsia (1).

^2 ^Significantly smaller (True Conjugate) or lower (Apgar) p < 0.05 compared to all other indications

^3 ^True conjugate significantly smaller compared to all other indications except fetal distress

For the indication 'malpresentation' (n = 55; 9,5%), 14 were due to breech (four had delay 1^st ^or 2^nd ^stage), 14 were due to twins (10 with transverse or breech in first neonate), 11 were due to compound presentation or arm prolaps and 16 were transverse lies.

The group 'other' (n = 34; 5,9%) included six cases done for a HIV positive mother, five women with extensive condylomata accuminata (unknown, but likely HIV positive), five women with a ruptured uterus during labor, two women had an elective caesarean for suspected macrosomy, two women with intra uterine growth retardation (IUGR) or placental insufficiency, two had a bad obstetric history, and for 12 cases no additional information was available.

The true conjugate is significantly smaller in the groups 'dystocia' and 'repeat caesarean section' when compared to the average of all other groups. The Apgar score is significantly lower after 5 minutes in the groups 'fetal distress', 'ante partum hemorrhage' and 'cord presentation', when compared to the average of all other groups (table 1.)

All caesarean sections done for 'dystocia' were intrapartum sections, as were those done for 'failed trial of scar', 'fetal distress' and 'cord presentation'. All caesarean sections recorded 'elective repeat' and 'preeclampsia' were antepartum sections. Caesarean sections done for 'malpresentation', 'antepartum hemorrhage' and 'other' can be either intra- or antepartum sections. There is no difference between the true conjugate for all true intrapartum indications (n = 306, mean true conjugate 9,7 cm) compared to all true antepartum indications (n = 141, mean true conjugate 9,6 cm).

Bilateral tubal ligation (BTL) was done in 114 patients (20%): 55 during the first (55/399), 10 during the second (10/108), 32 during the third operation (32/52) and in all women with fourth or fifth caesarean section (17/17).

Caesarean hysterectomy was performed in 10 women (1,7%): in four cases the indication for operation was uterine rupture, in three cases caesarean section was performed for antepartum hemorrhage and in three cases for dystocia. Indication for hysterectomy was postpartum hemorrhage from excessive uterine tearing, uterine atony not responding to conservative treatment and couvelaire uterus in placental abruption. Uterine scar rupture did not occur in those who had a 'trial of scar'. There was one caesarean hysterectomy after previous caesarean section. In this case the indication for the operation was antepartum hemorrhage resulting from placental abruption.

Finally, 37 neonates had an Apgar score < 7 after 5 minutes (6,4%). In nine of these cases fetal distress was the first indication for operation, indications for other caesareans were: antepartum hemorrhage (10), dystocia (eight), cord presentation (four), repeat caesarean (two), (pre) eclampsia (one) and others (three). In addition, 12 (2,1%) neonates were fresh stillbirths or died in theatre. Indication for caesarean section in these 12 cases were; antepartum hemorrhage (three), malpresentation (three), repeat caesarean (two), dystocia (two), fetal distress (one) and unknown (one). There were no maternal deaths.

## Discussion

The statistically significant lower true conjugate in women who underwent caesarean section for dystocia and repeat caesarean section (recurrent indications) as compared to the other (non recurrent) indications for caesarean section is interpreted in our study as some evidence for a valid reason to perform the operation. This is also supported by our relatively low caesarean section rate of 7,9%.

Unfortunately maternal height and neonatal birth weight, possible variables leading to dystocia, were not recorded. Furthermore, in this retrospective study comparisons between pelvic assessment from earlier preoperative vaginal examination and the internal pelvimetry results are not possible. Since data is only available from women delivered by caesarean and not for those who successfully achieved vaginal delivery, the true value of precise measurement is difficult to assess. However, although no records are available for the number of patients with successful trial of scar, the true conjugate of 'failed trial of scar' is comparable to the true conjugate of 'repeat caesarean' and significantly smaller when compared to all other indications for caesarean. This might indicate that patients selected for a trial of scar with a normal pelvis (true conjugate >9,0) deliver vaginally.

Dumont and colleagues report that of all caesarean sections, three-quarters are done for maternal indication. Furthermore, they suggest that in West Africa caesarean section for maternal indications (dystocia, previous caesarean, malpresentation, placenta praevia, abruptio placenta and (pre) eclampsia) is needed by 3,6–6,5% of pregnant women [19]. In our study the proportion of maternal indications among all caesarean sections is over 85% (even after excluding breech presentation), and the percentage caesarean sections done for maternal indications (6,7%) is at the higher range of figures from West Africa. The percentage of dystocia cases seems extremely high (one out of three caesareans) and the percentage of sections for fetal distress seems low (8.7%). In comparison, the national sentinel caesarean section audit from England, Wales and Northern Ireland [2], reported maternal indications to be the cause of > 60% of caesareans (dystocia 20,4%), while 23% are done for fetal indications and 12% due to breech or multiple pregnancy.

Clinical audit is seen as an essential component for improving the quality of care, but it is often found to be difficult to implement due to obstacles such as lack of time, resistance to change and lack of motivation [20]. Health care workers in African hospitals are no exception when it comes to these obstacles. In this rural hospital, the use of Delee Pelvimetry and the introduction of a caesarean section record book were found to be a simple and cheap method for the establishment of a continuous caesarean section audit by providing immediate feedback to the midwife and the doctor ('did I expect the true conjugate to be this small/large?'). Excluding patients whom underwent BTL or hysterectomy, in 89,6% of cases the true conjugate was measured. This might reflect a feeling on the part of the attending doctor that measuring is not always useful. Audit as described here, may increase the motivation to carry out the measurement.

Adadevoh *et al*. [4] advise that in all women undergoing laparotomy in their reproductive years the true conjugate should be measured and recorded for future reference. In addition, we recommend this measurement especially in those settings where very high CSR are observed. We hypothesize that in such settings the differences between recurrent and non-recurrent indications may not be statistically significant and measurements can thus help in reducing CSR.

## Conclusion

Improving the quality of obstetric care is an urgent priority worldwide. Audit can assist in this process by critical analysis of current practice and identification of substandard care factors. The use of DeLee's internal pelvimeter during caesarean section and keeping a 'caesarean section record book' are simple and cheap ways to introduce obstetric audit. This creates awareness, which may help in reducing unnecessary caesarean sections.

## List of abbreviations

BTL: bilateral tubal ligation

CPD: cephalo-pelvic disproportion

CSR: caesarean section rates

EOC: emergency obstetric care

HIV: human immunodeficiency virus

IUGR: intra uterine growth retardation

WHO: world health organization

## Competing interests

The author(s) declare that they have no competing interests.

## Authors' contributions

JvD initiated the study, analyzed the data and drafted the manuscript. TM initiated the use of DeLee pelvimetry and 'caesarean section record book' and assisted in revising the manuscript. VP assisted in study initiation and drafting of the manuscript. JvR assisted in analyses of the data and revised the paper critically for substantial intellectual content. All authors read and approved the final manuscript.

## Pre-publication history

The pre-publication history for this paper can be accessed here:



## References

[B1] Declercq E, Menacker F, MacDorman M (2005). Rise in "no indicated risk" primary caesareans in the United States, 1991–2001: cross sectional analysis. BMJ.

[B2] Thomas J (2001). The National Sentinel Caesarean Section Audit Report. Royal College of Obstetricians and Gynaecologists Clinical Effectiveness Support Unit.

[B3] Bauer O, Kingu R, Laussen T, Mbwana K (1998). Pelvic assessment and cephalo-pelvic disproportion in Central Tanzania. Int J Gynecol Obstet.

[B4] Adadevoh SWK, Hobbs C, Elkins TE (1989). The relation of the true conjugate to the maternal height and obstetric performance in Ghanaians. Int J Gynecol Obstet.

[B5] Burgess HA (1997). Anthropometric measures as a predictor of cephalopelvic disproportion. Trop Doct.

[B6] Liselele HB, Tshibangu CK, Meuris S (2000). Association between external pelvimetry and vertex delivery complications in African women. Acta Obstet Gynecol Scand.

[B7] Van Roosmalen J, Brand R (1992). Maternal height and the outcome of labor in rural Tanzania. Int J Gynecol Obstet.

[B8] Tbushi M, Ebrahim A, Moodley J, Shweni PM (1993). Vaginal delivery after previous caesarean section: is X-ray pelvimetry necessary?. Br J Obstet Gynaecol.

[B9] Van Loon AJ, Mantingh A, Elvira KS, Kroon G, Mooyaart EL, Huisjes HJ (1997). Randomised controlled trial of magnetic resonance pelvimetry in breech presentation at term. Lancet.

[B10] Philpott RH (1982). The recognition of cephalopelvic disproportion. Clin Obstet Gynaecol.

[B11] Armon P (1998). Cephalopelvic disproportion. Trop Doct.

[B12] Pattinson RC (2000). Pelvimetry for fetal cephalic presentations at term. Cochrane Database Syst Rev.

[B13] Meguid T (2002). Routine manual pelvimetries before labour in cephalic presentations in primigravidae: a personal opinion. PHC In Action.

[B14] Meguid T, Ikeakanam OTO, Petrova VA (2000). Lessons learned from two year of in-service-training for midwives at Onandjokwe hospital in northern Namibia. Int J Gynecol Obstet Suppl.

[B15] Wilder-Smith A (2003). Current Status of 'Essential Obstetric Care' activities internationally: a literature review. Trop Doct.

[B16] Holmgren G, Sjöholm L, Stark M (1999). The Misgav Ladach method for caesarean section: method description. Acta Obstet Gynecol Scand.

[B17] (2004). National Institute of Excellence, clinical guideline 13: Caesarean Section. http://www.nice.org.uk/CGO13niceguideline.

[B18] King M, King M (1993). The Surgery of Labour. Primary Surgery.

[B19] Dumont A, de Bernis L, Bouvier-Colle MH, Bréart G, MOMA study group (2000). Caesarean section rate for maternal indication in sub-Saharan Africa: a systematic review. Lancet.

[B20] Semple DM, Khaled K, Maresh MJA (2000). Monitoring quality of audit in obstetrics and gynaecology. Quality in Health Care.

